# Health of Louisville

**Published:** 1854-11

**Authors:** 


### HEALTH OF LOUISVILLE.

Up to this period the frosts are still delayed. The thermometer has not yet fallen below 38° in the city. The autumn has been one of the most delightful ever experienced in a region where autumn is proverbial for its delights. The city has continued healthy. Some cases of cholera were reported a few days since, but they were few in number, and all occurred within a very limited district in the upper end of the city, and within a few hours of each other. No cases have been reported since, and no apprehension of the spread of the disease was excited among the citizens.

